# The human testis-specific protein Y-linked (TSPY) is a male-specific cancer-testis antigen capable of eliciting significant immune responses and elimination of positive tumor cells in hepatocellular carcinoma

**DOI:** 10.1186/s13578-025-01432-8

**Published:** 2025-06-25

**Authors:** Tatsuo Kido, Yun-Fai Chris Lau

**Affiliations:** Laboratory of Cell and Developmental Genetics, Department of Medicine, San Francisco VA Health Care System, Institute for Human Genetics, University of California, 94121 San Francisco, US CA

## Abstract

The testis-specific protein Y-linked (TSPY) is a male-specific cancer-testis antigen specifically expressed in germ cells of the testis under normal conditions and various cancers, particularly in hepatocellular carcinoma (HCC), under oncogenic conditions. It binds to cyclin B and exacerbates the cyclin B-CDK1 phosphorylation of factors important for mitotic/meiotic divisions. To determine if such TSPY proliferative actions could contribute to various male-biases in liver cancer, TSPY transgene was expressed in an oncogene-induced preclinical mouse model of HCC, using the hydrodynamic tail vein injection strategy. The results showed that TSPY expression suppressed tumor cell growth at early stage but could evolve to resume oncogenic progression at late stage in this mouse model. Transcriptome and bioinformatic analyses demonstrated that significant immune and inflammatory responses were activated in early stage of the cancer, resulting in elimination of positive tumor cells. Significant TSPY antibodies were present in the sera of positive mice, similar to the presence of autoantibodies in the sera of patients positive for TSPY in their tumors. Flow cytometry and cellular protein fractionation analyses of positive tumor cells showed that TSPY protein could be mislocalized on the cell surface and likely be responsible for the humoral immunity. Additional studies demonstrated that TSPY peptides were produced and could form complexes with MHC-I molecules and be presented on the cell surface, thereby eliciting robust cytotoxic T cell responses and killing of positive tumor cells. Importantly these immune responses diminished and TSPY could exacerbate oncogenic growth at late stage. These findings suggest that as a male-specific cancer-testis antigen, TSPY is extremely immunogenic capable of eliciting robust immune and inflammatory responses at the early stage and is a significant candidate for development of immunotherapeutics, such as therapeutic cancer vaccine and antibody-drug conjugates, in treatments of hepatocellular carcinoma in men.

**Supplementary Information:**

The online version contains supplementary material available at 10.1186/s13578-025-01432-8.

## Introduction

The cancer-testis antigens (CTAs) are a large group of proteins that are exclusively expressed in the germ cells of the testis under normal conditions and in various tumor cells of many cancers under oncogenic conditions [1–3]. Most CTA genes are located on the X chromosome while the testis-specific protein Y-linked (TSPY) represents a male-specific CTA gene on the Y chromosome. They serve important functions in testicular/spermatogenic processes within the seminiferous tubules, which provides an immune privileged environment to protect the germ cells from systemic immune surveillance and preserves the male reproductive competency [1, 4–6]. When expressed in tumors, the CTAs could be involved in various oncogenic processes, including cell proliferation, apoptotic regulation, immune evasion, epigenetic modification, genome instability, angiogenesis, and metastasis [4, 7–11], thereby modifying the tumor cell physiology and behaviors. Due to their unique tumor-specific expression patterns outside their immune protective environment of the testis, the CTAs are being used as diagnostic and prognostic markers and proposed to be excellent targets for immunotherapy of various cancers [1, 3, 9, 11]. CTAs possessing oncogenic functions could be targets for functional/immunologic inhibitions while those possesses significant immunogenicity in their respective proteins could be targets for immunotherapy development, such as therapeutic cancer vaccines, in treatments of positive tumors. Hence, understanding the importance of the CTAs in these two aspects of mechanistic contributions to human cancer will be critical in the development of CTA-based therapeutic strategies for positive cancers.

TSPY is an ampliconic gene located on the critical region for gonadoblastoma locus on the Y chromosome (GBY) [12]. Recent complete sequencing of a human Y chromosome and those from 43 men of different ethnicities and geographical locations showed that most of the 2.8-kb TSPY gene is embedded in a highly conserved 20.3-kb unit tandem array, which is repeated 24–46 times on the short arm proximal to the centromere, representing as high as 42.5% of the protein-coding genes on this male-specific chromosome [13, 14]. GBY predisposes the dysfunctional gonads of patients with disorders of sex development (DSD, e.g. XY females and hermaphrodites) harboring residual Y sequences, including TSPY, to gonadoblastoma development at extremely high frequency (i.e. >67%) [15]. Gonadoblastoma is a benign germ cell tumor and the precursor for more aggressive germ cell tumors, such as dysgerminoma and testicular seminoma [16]. TSPY is normally expressed in gonocytes in the embryonic testis [17] and spermatogonia and spermatocytes in the adult testis and contributes to the replication of the gonocytes and spermatogonia and promotes meiotic divisions during spermatogenesis in adult testis [6, 18]. TSPY binds to cyclin B and exacerbates the cyclin B-CDK1 phosphorylation of factors important for mitotic/meiotic divisions [19, 20]. It is also expressed in various germ cell tumors, including gonadoblastoma and testicular germ cell tumors as well as numerous somatic caners, such as prostate cancer and hepatocellular carcinoma [21, 22]. Overexpression of TSPY promotes cell proliferation by abbreviating the G_2_/M transitions in tumor cells [23]. TSPY interacts with the androgen receptor (AR), and AR variants and stimulates AR/AR-variant transactivation of target genes in a ligand dependent and independent manners respectively [24]. Significantly, TSPY is also an AR responsive gene [20, 25], thus TSPY and AR form a positive feedback loop in male sex hormone responsive physiology/oncogenesis [20]. Various studies supported the role of TSPY as the proto-oncogene for gonadoblastoma locus on the Y chromosome [20].

Among the various positive human cancers TSPY expression is the highest in hepatocellular carcinoma (HCC) (33%), followed by lung adenocarcinoma (17%), head and neck cancer (11%), bladder cancer (10%), and kidney cancer (6%). The TSPY protein is co-localized with specific tumor markers on the tumor cells, but not adjacent non-tumor cells [21, 26]. As a putative proto-oncogene for GBY and cell cycle regulator, TSPY expression in somatic cancers could play key roles in various male biases in incidence, progression, and treatment responses among the positive tumors, particularly HCC [21]. Thus, it could be a key CTA candidate for functional inhibition in various sexually dimorphic/male-specific cancers [27, 28]. To address such possibilities, we have examined the effects of TSPY expression in a transgenic mouse model of HCC using the hydrodynamic tail vein injection strategy. Our results, however, showed that TSPY protein possesses extremely high immunogenicity and can elicit robust immune responses from the hosts, resulting in elimination of positive tumor cells at the early stage. Additional studies indicated that TSPY protein/peptide could be mislocalized on the cell surface and/or form complexes with MHC-I molecules and activate the cellular immune responses. It could also elicit significant humoral immune responses, resulting in the synthesis of specific antibodies in the sera of the hosts. Residual tumor cells, however, could evade such tumor immune surveillance and evolve to resume aggressive oncogenic growth at later stage, resulting in poor survival of the host animals. Transcriptome analysis demonstrated the activation of various immune cells associated with both humoral and cellular immune responses/processes at early stage, which diminished at late stage when the tumor resumed aggressive growth. Thus, TSPY possesses dual functions as an immunogenic CTA eliciting immune surveillance at early stage and promoting oncogenic progression at late stage in HCC.

## Materials and methods

### Plasmids

The pT3-EF1α-based HA-myr-Akt vector (designated as pT3-AKT^myr^), the pT2-Caggs-based NRasV12 vector (designated as pT2-NRAS^V12^), the pT3-EF1α empty vector, and the pCMV/sleeping beauty transposase vector (designated as pCMV-SB) were kindly provided by Dr. Xin Chen (University of Hawaii) [29]. The pT3-EF1α-based EGFP expression vector, pT3-EGFP, and the TSPY-IRES-EGFP cassette, which enables simultaneous expression of FLAG-tagged TSPY and EGFP genes via an internal ribosome entry site, were generated previously [24, 30]. The TSPY-IRES-EGFP cassette was inserted into the pT3-EF1α plasmid using the Gateway LR Clonase II system (Thermo Fisher Scientific, Waltham, MA) to create the pT3-EF1α-TSPY-IRES-EGFP vector (designated as pT3-TSPY-EGFP).

## Mouse hepatocellular carcinoma model using hydrodynamic tail vein injection

Hydrodynamic tail vein injection was performed as described previously [29, 31]. In brief, the 5- to 6-weeks-old male FVB mice (approximately 20 g body size) were randomly divided into 5 groups of 3 animals each for groups 1 to 5. The plasmid mixture as described in Table 1 were diluted in 2 ml saline (0.85% NaCl), sterilized through 0.2 μm filter and injected into the lateral tail vein of a recipient mouse in 7 s. Animals were monitored twice weekly for tumor growth, harvested at 6 weeks or 9 weeks post-injection for analyses.

**Table 1:** Composition of plasmid solutions for hydrodynamic tail vein injection

Plasmids	Group 1	Group 2	Group 3	Group 4	Group 5	Group 6	Group 7
Group Names	AKT + NRAS + EGFP @6week	AKT + NRAS + TSPY @6week	TSPY @6week	Control @6week	AKT + NRAS + TSPY @9week	AKT + NRAS + TSPY +B2mKO	AKT + NRAS + TSPY +ControlKO
Abbreviated Names	EGFP-6wk	TSPY-6wk	TSPY alone	Control	TSPY-9wk	B2mKO	ControlKO
*pCMV-SB*	2	2	2	-	2	2	2
*pT3-AKT* ^*myr*^	10	10	-	-	10	10	10
*pT2-NRAS* ^*V12*^	10	10	-	-	10	10	10
*pT3-EGFP*	30	-	-	-	-	-	-
*pT3-TSPY-EGFP*	-	30	30	-	30	30	30
*pQCi-mB2m-sgRNA*	-	-	-	-	-	20	-
*pQCi-control-sgRNA*	-	-	-	-	-	-	20

(µg/2 mL saline)

To investigate the role of the MHC-I complex in the TSPY-dependent immune response, the β2-microglobulin (B2m) gene, coding for a key component of MHC-I, was knocked out by co-injection of the CRISPR-based plasmid pQCi-mB2m-sgRNA or a control sgRNA plasmid [32], obtained from the Addgene repository (Watertown, MA) (Table 1, Group-6 and Group-7 respectively). The mB2m-sgRNA targets the sequences 5’- TCGGCTTCCCATTCTCCGGT-3’ in exon 2 of the mouse B2m gene. Transfection of this special plasmid and sgRNA in the cultured mouse T-lymphoblast EL4 cells resulted in knockout of the endogenous B2m gene in approximately 38% of transfected mouse cells [32]. The efficiency of in vivo knockout in the liver of mouse using hydrodynamic tail vein injection varies depending on the reagents and experiment conditions and could vary from negligible to up to 40% [33].

Gross images of the raw tissues were captured in the bright field and fluorescence mode with a Leica MZFLIII fluorescence microscope and Zeiss AxioCam MRc digital imaging system. All experimental procedures were approved by the Institutional Animal Care and Use Committee in accordance with the NIH Guide for Care and Use of Laboratory Animals.

## Immunohistochemistry and Immunofluorescence

Immunohistochemistry and immunofluorescence were performed as described previously [34, 35], following fixation with 4% paraformaldehyde in PBS and paraffin embedding using a standard protocol. The following primary antibodies were used; anti-B2M rabbit polyclonal antibody (1:800 dilution, Proteintech Group, Rosemont, IL), anti-CD3 rat monoclonal antibody (1:100 dilution, clone CD3-12, Bio-Rad, Hercules, CA), anti-FLAG tag mouse monoclonal antibody (1:800 dilution, clone M2, Sigma-Aldrich, St. Louis, MO), and anti-TSPY mouse monoclonal antibody (1:100 dilution, clone SF-7, Santa Cruz Biotechnology, Dallas, TX). Immunoreactive signals were visualized using either VECTASTAIN Elite ABC-HRP kit (Vector Laboratories, Burlingame, CA) or fluorophore-conjugated secondary antibodies; Alexa Fluor 594 conjugated anti-mouse IgG (red), Alexa Fluor 488 conjugated anti-rabbit IgG (green), or Alexa Fluor 488 conjugated anti-rat IgG (green) (Thermo Fisher Scientific, Waltham, MA). Nuclear staining was performed using hematoxylin (Vector Laboratories) or 4’,6-Diamidine-2’-phenylindole dihydrochloride (DAPI) (Roche, Indianapolis, IN). Detection of apoptotic cells in tissue sections was carried out using the terminal deoxynucleotidyl transferase dUTP nick end labeling (TUNEL) assay with the ApopTag Peroxidase In Situ Apoptosis Detection kit (Sigma-Aldrich). Bright-field and fluorescent images were acquired using an AxioCam digital camera and an image acquisition and analysis workstation (Zeiss Microscopy, White Plains, NY). The detailed information of antibodies is listed in Supplementary Table S1.

## RNA-seq transcriptome analysis

Liver tumor foci were dissected and pooled for each mouse at 6 or 9 weeks post-hydrodynamic injection. Total RNA was isolated from tumor foci using TRIZOL-Plus RNA isolation kit (Thermo Fisher Scientific). One µg of total RNA from each biological triplicate sample was used for library preparation with the KAPA mRNA HyperPrep Kit (Roche) and sequenced on a NextSeq 500 sequencer (Illumina, San Diego, CA), as described previously [30, 36, 37]. Low-quality reads were trimmed using Trimmomatic (version 0.38.1) [38]. The processed reads were then aligned to the Ensembl GRCm39 (release M27) mouse reference genome using STAR software (version 2.7.3a) [39] and converted to read-counts per gene using the featureCounts software (version 2.0.0) [40]. RNA-seq data from mice 20 days post hydrodynamic injection of the pT3-EF1α empty vector served as control data for transcriptomic analyses [31].

Normalization and differential gene expression analyses were performed using a TCC/EdgeR software package [41]. Genes representing changes with TCC/edgeR software analysis FDR < 0.05, Student’s t-test p-value < 0.05, Log _2_ (gene expression level) > 2.7, and|Log _2_ (fold change)| > 1.2 were considered as differentially expressed genes (DEGs). Gene enrichment analyses for associated biological processes were performed using DAVID Bioinformatics Resources (version 2024) [42], with processes achieving an FDR < 0.05 considered statistically significant. The list of marker genes for immune cell profiling was obtained from the Cell Marker 2.0 database [43] and the Immune Cell Markers Selection Tool (Bio-Rad). The datasets generated and/or analyzed during the present study are available upon request.

## Flow cytometry analysis

Huh7-tetON-TSPY-EGFP cells, which co-express FLAG-tagged TSPY and EGFP genes under doxycycline control, and Huh7-tetON-EGFP cells, which express EGFP alone in a doxycycline dependent manner, were generated as described previously [30]. Cells were seeded at 3 × 10^5^ cells per 10 cm culture dish in DMEM containing 10% fetal bovine serum (TET approved FBS, BioWest, Bradenton, FL) and cultured for 48 h in the presence of 1 𝜇g/ml doxycycline to induce transgene expression. Flowcytometry was performed using a standard protocol. In brief, cells were harvested in FACS buffer (phosphate-buffer saline (PBS), 3mM ethylenediaminetetraacetic acid (EDTA), 5% FBS, 0.1% sodium azide), and blocked with 50 𝜇g/ml eBioscience Fc blocker (Fisher Scientific) for 20 min on ice. Following blocking, cells were incubated with primary antibodies at 4℃ for 30 min. After washing with Biolegend Cell Staining Buffer (Fisher Scientific), cells were incubated with the secondary antibody at 4℃ for 30 min, either Alexa Fluor 647-conjugated anti-mouse IgG (Thermo Fisher Scientific) for mouse monoclonal antibodies or Alexa Fluor 647-conjugated anti-rabbit IgG (Thermo Fisher Scientific) for rabbit antiserum. Cells were subsequently fixed with 2% paraformaldehyde in PBS at room temperature for 15 min and rinsed with ice-cold PBS. Flowcytometry of stained cells were performed on a FACS Aria IIu (BD Biosciences) at the San Francisco VA Medical Center Flow Cytometry Core Facility, and the data was analyzed using FlowJo Software 10.10 (BD Biosciences).

## Cell surface protein fractionation

Huh7-tetON-TSPY-EGFP cells were seeded at 8 × 10^5^ cells per 15 cm culture dish and cultured for 48 h in the presence of 1𝜇g/ml doxycycline. After washing with PBS, the cell surface proteins were isolated using the Pierce Cell Surface Protein Biotinylation and Isolation kit (Thermo Fisher Scientific) [44, 45]. In brief, live cells were incubated with sulfo-NHS-SS-biotin at room temperature for 10 min, rinsed with ice-cold Tris buffer saline (TBS) to remove unreacted sulfo-NHS-SS-biotin, and lysed in 500 𝜇l of lysis buffer. The lysate was incubated with NeutrAvidin agarose beads to capture biotinylated surface proteins for 30 min at room temperature, followed by centrifugation to remove the flow-through. The flow-through was retained as the cytoplasmic fraction. Biotinylated surface proteins were eluted in 225 𝜇l of elution buffer containing 10 mM dithiothreitol (DTT) for de-biotinylating and incubated at room temperature for 30 min. The samples were subsequently analyzed by western blotting.

## Western blotting

Tumor samples weighting 140 mg were homogenized in 140 μL ice-cold PBS containing cOmplete Mini Protease Inhibitor Cocktail (Roche Diagnostics), followed by the addition of 280 μL of 2x SDS PAGE sample buffer. After sonication for 20 s using a W185-F sonicator (Heat Systems-Ultrasonics, Plainview, NY), tumor lysates were incubated at 96℃ for 10 min to ensure denaturation, centrifuged at 16,000 ×g for 10 min at 4℃, and the supernatant was collected for analysis.

The human embryonic kidney 293T cells were cultured in DMEM supplemented with 10% FBS in 24-well plate (1.9 cm^2^ surface area) at 1/6 confluency. The cells were transfected with 0.5 µg of the pT3-EGFP or pT3-TSPY-EGFP plasmid using the X-tremeGENE9 transfection reagent (Roche), following the manufacturer’s instructions. After 48 h of culture, the cells were lysed in 100 µl of SDS-PAGE sample buffer and incubated at 96℃ for 10 min to achieve denaturation. Western blotting of protein lysates (from cell cultures and mouse tissues) were performed as previously described [46], using anti-TSPY rabbit antibody (1:4000 dilution, in house) [47], anti-B2M rabbit polyclonal antibody (1:2000 dilution, Proteintech Group), anti-βactin mouse monoclonal antibody (clone AC-15, 1:8000 dilution, Sigma-Aldrich), anti-GFP goat polyclonal antibody (1:2000 dilution, Abcam, Waltham, MA), or serum from Group-1 or Group-2 mice (1:2000 dilution). Immunoreactive signals were visualized by IRDye680 conjugated anti-mouse IgG antibody or IRDye800 conjugated anti-rabbit IgG antibody and recorded with the Odyssey system (LI-COR, Lincoln, NE).

## Results

### TSPY suppresses tumorigenic growth in an oncogene-induced HCC mouse model

**Fig. 1:**
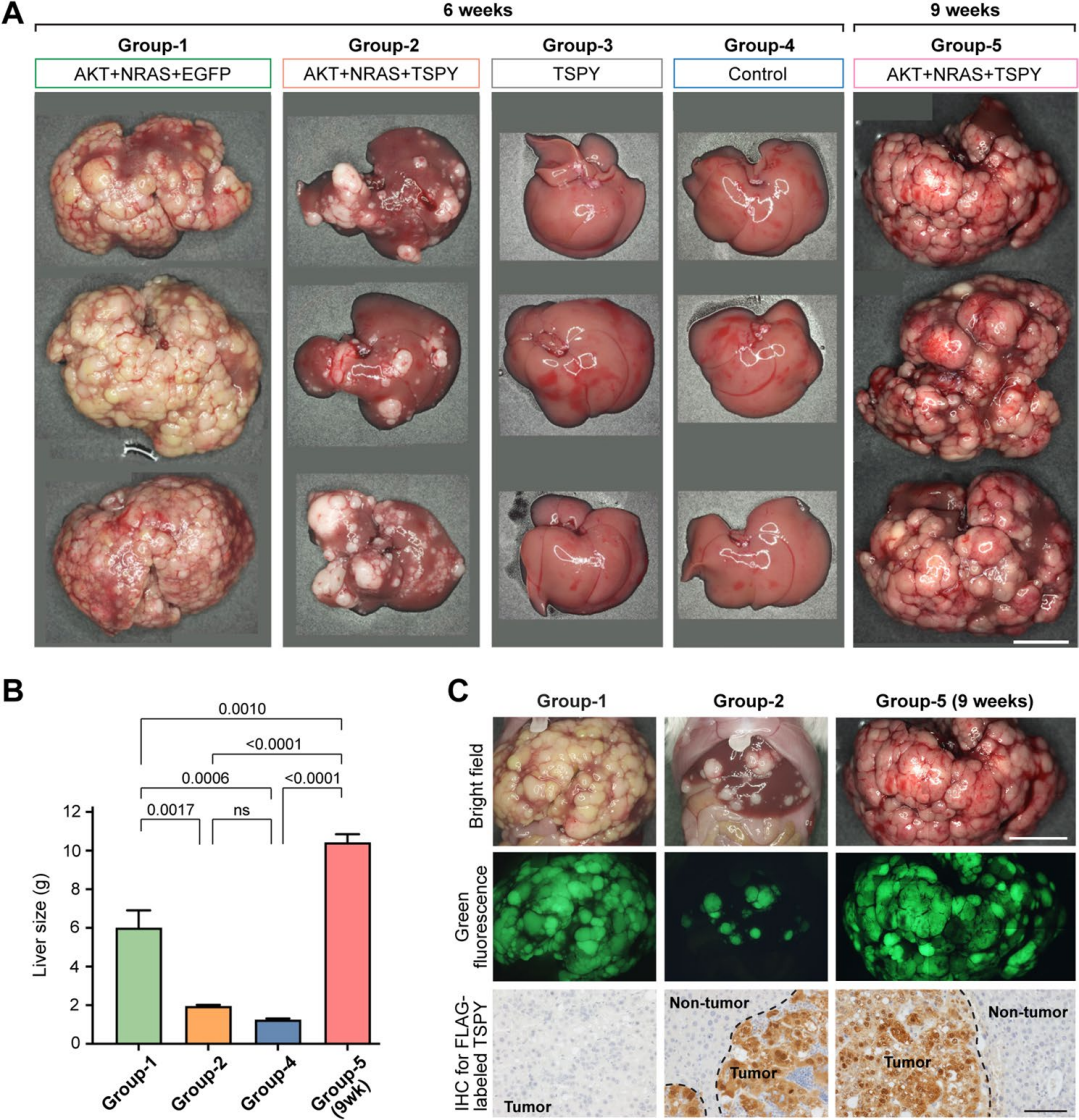
Effects of TSPY expression in tumors induced by AKT + NRAS oncogenes in the livers of mice using hydrodynamic tail vein injection strategy. (**A**) Group-1 represents tumors induced by oncogenes without TSPY at 6 weeks post-injection (AKT + NRAS + EGFP@6week). Group-2 represents tumors with TSPY expression (AKT + NRAS + TSPY@6week); Group-3 represents livers with only TSPY transgene injection (TSPY alone@6week). Group-4 represents non-injected controls (no injection control@6week); and Group-5 represents tumors with TSPY at 9 weeks post-injection (AKT + NRAS + TSPY@9week). The results showed that TSPY expression suppressed tumor growth at 6 weeks, but tumors could resume oncogenic progression to similar size at 9 weeks as those without TSPY expression in Group-1 at 6 weeks. (**B**) The liver sizes of mice of the four groups of mice injected with various gene combinations, indicating statistically significant large tumors in Group-1 and Group-5 mice, but not Group-2 and Group-4 mice (See Table 1). (**C**) Tumors induced by oncogenes (top row) co-expressed with the green fluorescent protein (middle row) and TSPY detected by immunohistochemistry in corresponding tumor sections (bottom row), indicating EGFP is a reliable fluorescent tracer for tumorigenesis (Group-1) and TSPY expression on positive tumors (Group-2 and − 5). Scale bar = 1 cm in A and the top row of C, 100 μm in the bottom row of C

The high levels of TSPY expression and poor prognosis in positive HCC patients raise the possibility of its contribution(s) to the male-biases in this type of liver cancer [21, 30]. To explore the potential pro-oncogenic actions of an aberrantly expressed TSPY in hepatocarcinogenesis, we had used the well-established hydrodynamic tail vein injection strategy to express a human TSPY transgene in an oncogene-induced mouse model of HCC [29, 37]. Hydrodynamic tail vein injection of DNA of the constitutively active oncogenes, i.e. myristoylated Akt (Akt^myr^) and NRAS with G12V substitution (NRAS^V12^), and the Sleeping Beauty (SB) transposase gene primarily delivers the DNA to the liver of the host, where most hepatocytes will initially incorporate and express the transfected genes transiently. The SB could mediate an integration(s) of the transfected DNAs into the genome of selected cells, which undergo oncogenic transformation and give rise to tumor foci and eventually HCC within a few weeks [29]. To evaluate the effects of TSPY in this HCC model, we co-injected the oncogenes together with various gene constructs in different combinations to groups of mice with (1) EGFP gene alone as a tracer (Group-1 = AKT + NRAS + EGFP@6week, referred to as EGFP-6wk), (2) TSPY-EGFP co-expression construct (Group-2 = AKT + NRAS + TSPY@6week, referred to as TSPY-6wk), (3) TSPY-EGFP gene alone without oncogenes (Group-3 = TSPY alone), and (4) sham saline/no injection (Group-4 = noninjected control) (Table 1). Tumor growth in the livers of the injected mice were monitored twice weekly by palpation and were harvested at 6 weeks post injection. They were initially examined with standard necropsy and pathologic assessment of the tumor sizes and morphology. Our results showed that significant tumors developed in the livers of the oncogene-alone Group-1 mice, necessitating an experimental end point at 6 weeks post-injection, as previously demonstrated [29]. Inclusion of TSPY-EGFP construct in Group-2 mice showed a significant reduction of tumor size while those injected with TSPY-EGFP alone (Group-3) or control (Group-4) showed no tumor in the respective livers (Fig. 1A & B), suggesting that TSPY alone is incapable of inducing tumor development while TSPY co-expression with the oncogenes led to a repression/reduction of tumorigenic growth in this mouse HCC model. These observations are unexpected and in contrast to the presumed pro-oncogenic functions of TSPY. Similar repression/reduction of TSPY-positive tumors were observed in repeated experiments and those with other oncogene combinations, including MYC +  β-catenin [48] and MYC + AR-V7 [31] (data not shown), suggesting that TSPY co-expression was responsible for consistent inhibition of oncogene-induced tumorigenesis in the liver using the hydrodynamic tail vein injection strategy.

Since mice co-expressing TSPY-EGFP with the oncogenes harbored significantly smaller tumors in their livers, selected mice were housed further and monitored similarly. At 9 weeks post-injection, these mice reached an experimental end point with tumor sizes similar to/larger than those of Group-1 mice at 6-weeks (Fig. 1A & B). Thus, they were harvested at 9 weeks and designated as Group-5 (AKT + NRAS + TSPY@9week, referred to as TSPY-9wk). Our observations suggest that TSPY initially suppressed tumor growth at early stage, but its suppressive effects diminished, and residual tumor cells resumed growth at a later stage. Fluorescence microscopy showed that the tumor foci in Group-1, -2 and -5 were positive for the EGFP fluorescence, which were also positive for TSPY protein in both Group-2 and -5 mice but not Group-1 mice by immunohistochemistry (Fig. 1C). Thus, EGFP was used successfully as a tracer for tumor growth, and its expression could be directly linked to that of TSPY in the TSPY-EGFP bicistronic transgene in the tumors of both Group-2 and -5 mice. Immunohistochemistry showed that indeed the green fluorescent tumor foci were positive for TSPY in Group-2 and -5 mice, but not those with the oncogenes alone in Group-1 mice (Fig. 1C, lower row).

### TSPY-mediated suppression of tumor growth via activation of immune responses in the hosts

To gain a more comprehensive view of the tumors at various conditions, tumor tissues were dissected with the aid of the co-expressed EGFP tracer (Fig. 1C) and analyzed with RNA-Seq transcriptomes in biological triplicates as before [30, 31, 37]. Initially the differentially expressed genes (DEGs) at each group were compared to those of control livers (i.e. Group-1, 2 and 5 versus Group-4). Significant numbers of DEGs were identified A) showing 2528 up and 831 down genes in Group-1, 2964 up and 888 down genes in Group-2, and 2668 up and 907 down genes in Group-5, as compared to Group-4, Supplementary Table S2A-C.

**Fig. 2:**
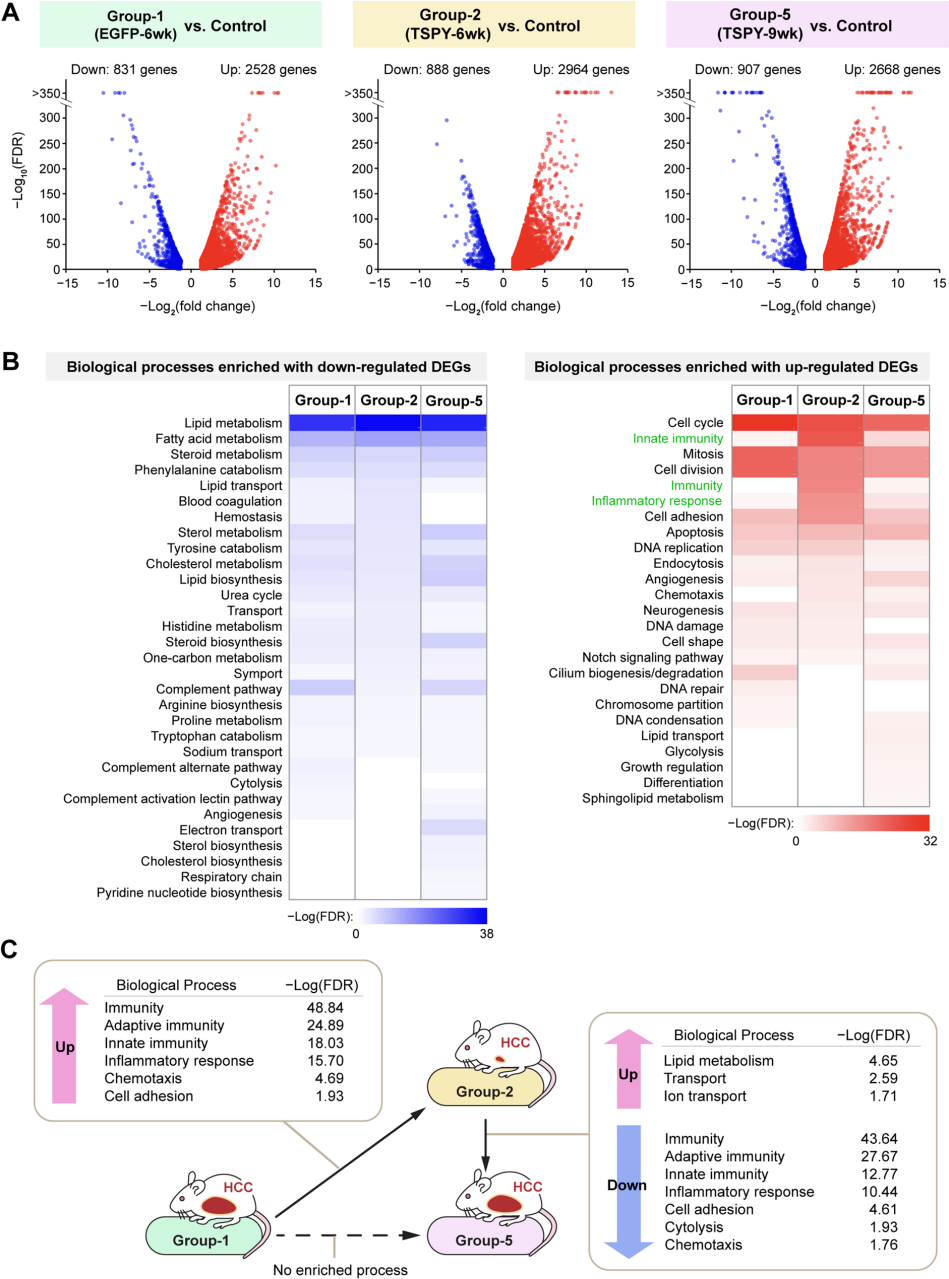
Transcriptome and pathway analyses of differentially expressed genes among various oncogene-induced tumors with and without TSPY expression. (**A**) Volcano plots of differentially expressed genes in Group-1 (AKT + NRAS + EGFP@6week, labeled as EGFP-6wk) versus Control (left), Group-2 (AKT + NRAS + TSPY@6week, labeled as TSPY-6wk) versus Control (middle) and Group-5 (AKT + NRAS + TSPY@9week, labeled as TSPY-9wk) versus Control (right). (**B**) Gene ontology enrichment analyses of differentially down-regulated genes (blue, left) and up-regulated genes (red, right) in respective tumor samples as compared to the control liver. Various metabolic processes, e.g. lipid metabolism and fatty acid metabolism, were down-regulated in the tumor samples (left panel, blue). Cell cycle, mitosis and cell division biological processes were significantly up-regulated in all tumors, as expected in oncogenic growth (right panel). Importantly, innate immunity, immunity and inflammatory responses (right panel, green labeled) were up-regulated primarily in Group-2 (TSPY-6wk) tumors but not those of others, suggesting that robust immune and inflammatory responses were extremely active in the Group-2 tumors. (**C**) Gene ontology enrichment analysis of differentially expressed genes between tumors of Group-1 (EGFP-6wk) and Group-2 (TSPY-6wk), showing significant up-regulation of immunity, adaptive immunity, innate immunity and inflammatory responses in Group-2 tumors. Similar analyses of differentially expressed genes between Group-2 and Group-5 (TSPY-9wk) showed such immune and inflammatory responses were down-regulated in Group-5 tumors, while no enriched processes were identified between Group-1 and Group-5 tumors

Gene Ontology analyses using the DAVID bioinformatics resource [49] showed that most down-regulated genes were significantly (*p*-value < 0.05) enriched in lipid, fatty acid and steroid metabolism pathways (Fig. 2B, left), as expected from the oncogenic conditions. The up-regulated genes were enriched in cell cycle, mitosis and cell division signaling pathways across the tumor samples (Fig. 2B, right). Noticeably, various innate immunity, immunity and inflammatory response processes were up-regulated primarily in tumors of the Group-2 (TSPY-6wk) mice but not those for the Group-1 (EGFP-6wk) nor the Group-5 (TSPY-9wk) mice (Fig. 2B, right, green labels), suggesting that significant immune and inflammatory responses were present in the tumor microenvironment in Group-2 mice with TSPY expression at early stage of the hepatic oncogenesis.

Gene ontology enrichment analysis of the differentially expressed genes (DEGs) [42, 49] between the Group-2 (TSPY-6wk) and Group-1 (EGFP-6wk) tumors identified various signaling pathways and biological processes present within the DEGs between the two tumor samples. The results showed that the Group-2 tumors possessed significant elevated expression of pathways in immunity, adaptive immunity, innate immunity, inflammatory response, chemotaxis, and cell adhesion, as compared to Group-1 tumors without TSPY (Fig. 2C, Supplementary Table S3A). Comparison between the tumors from Group-2 (TSPY-6wk) and Group-5 (TSPY-9wk) mice showed that such immune responses in Group-2 (TSPY-6wk) tumors were significantly down-regulated with a moderate resumption of lipid metabolism and ion transport in Group-5 (TSPY-9wk) tumors (Supplementary Table S3B). No significant change in biological processes was identified between Group-1 (EGFP-6wk) and Group-5 (TSPY-9wk) tumors (Supplemental Table S3C).

**Fig. 3:**
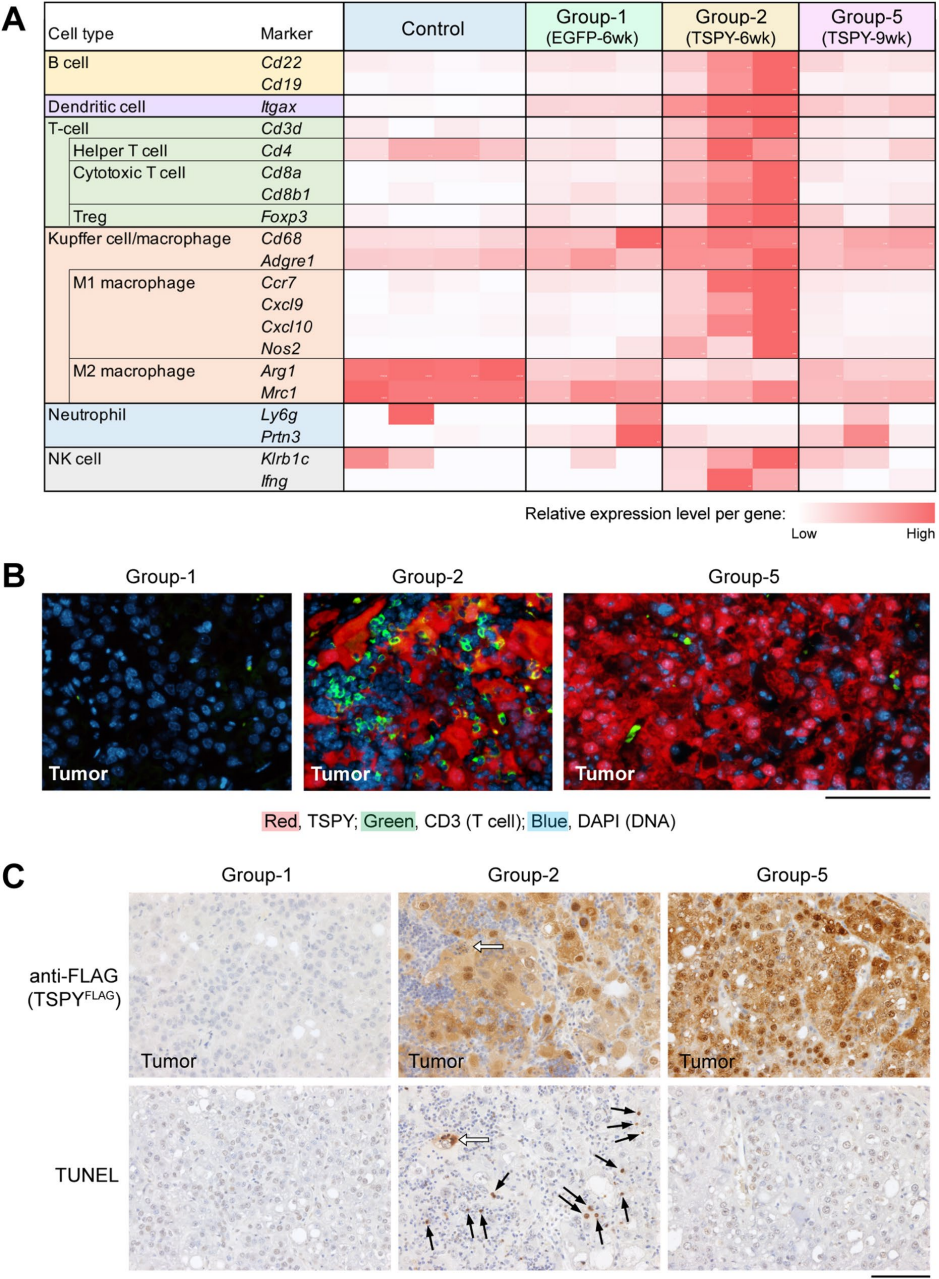
Significant activation of various types of immune cells involved in immune and inflammatory responses and induction of cell death in Group-2 (TSPY-6wk) tumors. (**A**) The relative expression levels of specific markers for various immune cells showed B cell, dendritic cell, T cell subtypes, M1 macrophage, and NK cells were highly expressed in Group-2 (TSPY-6wk) tumors, but not the others, suggesting that the humoral and cellular immune and M1 macrophage-mediated inflammatory responses were present and highly activated in these TSPY-positive tumors of Group-2 at 6 weeks. (**B**) Immunofluorescence of the general T cell CD3 marker showed that significant T cell infiltrations were present in Group-2 (TSPY-6wk) tumors. (**C**) Immunohistochemistry (top row) and TUNEL staining (bottom row) confirmed the expression of TSPY protein (FLAG-labeled) in both Group-2 and Group-5 tumors, which was associated with positive TUNEL staining in Group-2 tumor only (arrows) but not those of the others, suggesting that TSPY expression elicited robust immune and inflammatory responses leading to tumor cell apoptosis in Group-2 mice at early stage. Open arrows indicate a likely apoptotic cell in Group-2 tumor. Scale bar = 100 μm in B and C

Using the expression levels of specific markers for various immune cells [43, 50], significant activation of B cells, dendritic cells, T cell subtypes and Kupffer cell/macrophage involved in various humoral and cellular immune responses were observed in the tumors of Group-2 (TSPY-6wk) animals, but not those of the others (Fig. 3A). Importantly, the pro-inflammatory M1 but not M2 macrophages were highly activated in Group-2 (TSPY-6wk) mice, indicating that pro-inflammatory responses were active in TSPY-expressing tumors at this stage, supporting the postulation that TSPY expression in tumor cells elicited robust immune and inflammatory responses at the early stage of hepatocarcinogenesis in the host animals. Such immune and inflammatory responses could subside, and the residual TSPY-positive tumor cells could resume growth at later stage to large tumors, similar to those without TSPY at 6 weeks (Group-1) post-injection in this mouse model.

Using the general T cell marker CD3 [51], we demonstrated that significant T cell infiltration was presented in Group-2 (TSPY-6wk) tumors as compared to those of Group-1 (EGFP-6wk) and Group-5 (TSPY-9wk) (Fig. 3B). Similarly, TUNEL staining showed that relatively higher number of apoptotic cells were present in the tumors of the Group-2 (TSPY-6wk) mice (Fig. 3C). These observations support the postulation that immune and inflammatory responses could be responsible for the tumor cell elimination and smaller tumor sizes in the Group-2 (TSPY-6wk) mice.

### Sera from mice with TSPY-positive tumors harbor TSPY antibodies

**Fig. 4:**
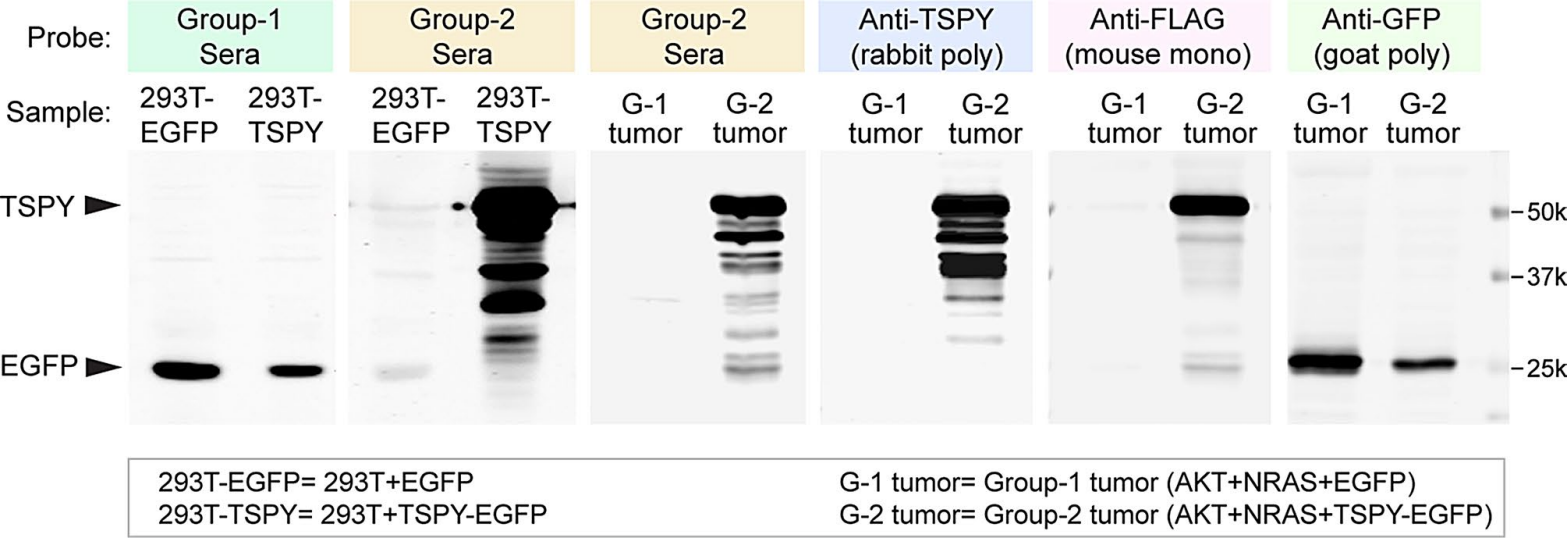
Detection of anti-TSPY antibodies in the sera of positive mice. Protein lysates of 293T cells transfected with either EGFP (293T-EGFP) or TSPY-EGFP (293T-TSPY) and tumors from either Group-1 (G-1) or Group-2 (G-2) mice were analyzed with western blots using sera from Group-1 and Group-2 mice. The serum of individual Group-1 mice detected only the EGFP protein in 293T cells transfected with EGFP or TSPY-EGFP vector while those from Group-2 mice detected TSPY protein only in 293T cells transfected with TSPY-EGFP construct or Group-2 mice harboring TSPY-positive tumors. Left 2 panels (293T cells), right 4 panels, mouse tumor protein lysates. A rabbit polyclonal antibody against TSPY (Anti-TSPY rabbit poly) and a monoclonal antibody against the FLAG epitope (Anti-FLAG mouse mono) were used as references for detection of various fragments and full length TSPY protein respectively. A goat anti-GFP polyclonal antibody (Anti-GFP goat poly) was used to detect the EGFP protein in the tumors (rightmost panel). Detection of various TSPY fragments in both transfected 293T cells and positive tumors suggested that the TSPY protein was degraded/processed by proteasomal mechanisms in both cultured 293T cells and tumors of positive mice

In clinical studies, TSPY autoantibodies were frequently detected in the sera of male HCC patients [52]. To determine if such positivity was present in mice bearing TSPY positive tumors, sera were collected from various groups of mice (Table 1) and analyzed for their reactivities in western blots against lysates of HEK293T cells (negative for TSPY) transfected individually with TSPY-EGFP or EGFP control construct. The results showed that sera from *all* mice in Group-2 (TSPY-6wk) and Group-5 (TSPY-9wk) reacted intensively against the TSPY protein while those from the Group-1 (EGFP-6wk) mice did not (Fig. 4, left, 293T), suggesting that TSPY antibodies were present in the sera of mice harboring TSPY-positive tumors, similar to the sera of patients [52]. Western blotting of tumor lysates with the Group-2 sera showed the presence of TSPY protein in Group-2 (G-2) but not Group-1 (G-1) tumors (Fig. 4, right Tumor). The positive bands in the Group-2 tumors were confirmed with a TSPY specific rabbit polyclonal antibody. Using an anti-FLAG antibody, we demonstrated that the largest band likely corresponded to full-length TSPY protein harboring a FLAG epitope at its N-terminus, while the smaller bands positive for both Group-2 sera and rabbit TSPY antibody could represent degraded or proteasomally processed TSPY fragments in both the Group-2 tumors and transfected HEK293T cells. Similar experiments showed that sera from Group-5 were capable of detecting TSPY in both transfected HEK293T cells and tumors, suggesting that TSPY-specific antibodies persisted in these mice. Our findings suggested that antibodies against TSPY were synthesized in mice bearing TSPY-positive tumors, similar to those observed in selected male HCC patients [52]. We surmise that such TSPY antibodies could mediate various humoral immune responses [53, 54], including activating the complement system and antibody-dependent cell-mediated cytotoxicity (ADCC), thereby eliminating TSPY-positive tumor cells in the host.

### Detection of TSPY epitopes on the surface of tumor cells by flow cytometry

**Fig. 5:**
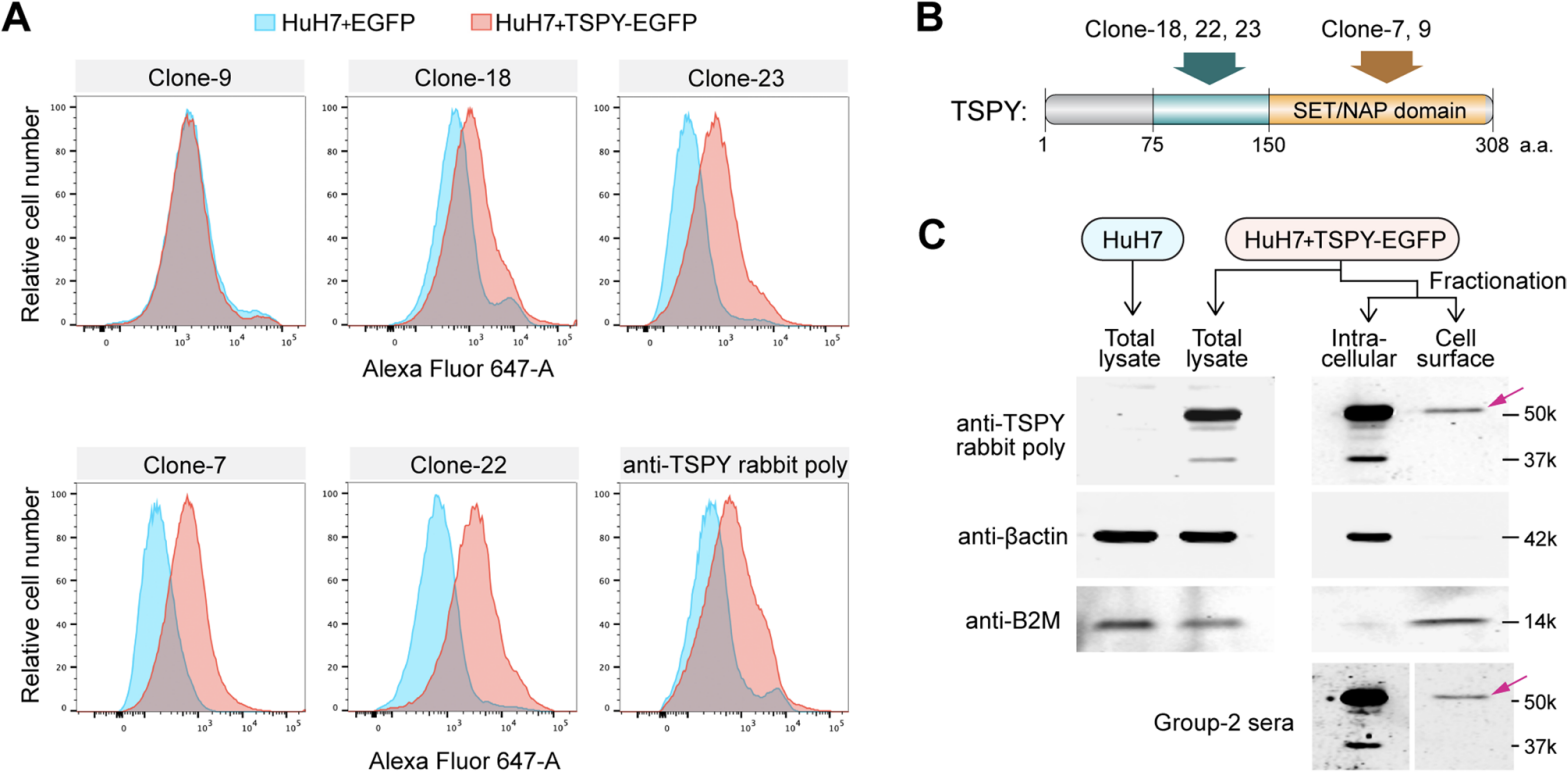
Demonstration of TSPY protein location on cell surface using flow cytometry and cell fractionation analyses on HCC HuH7 tumor cells. HuH7 cells were transfected with either an EGFP or a TSPY-EGFP vector, representing TSPY negative and positive cells respectively. (**A**) They were analyzed with flow cytometry for cell surface proteins using a panel of 9 TSPY monoclonal antibodies (5 presented here) showing various degrees of bindings to the surface of TSPY-positive cells (pink) and shifting to the right from the negative cells (blue). A rabbit TSPY polyclonal antibody was used as a reference (bottom right). (**B**) Diagrammatic illustration on the approximate locations of the epitopes of respective monoclonal antibodies along the TSPY protein. (**C**) HuH7 cells negative (blue label) and positive (red label) for TSPY were fractionated into intracellular and cell surface components and analyzed with western blotting with antibodies against TSPY (top row), anti-βactin (an intracellular marker, middle row) and anti-β2-microglobulin (B2M a cell surface marker, bottom row), showing the presence of TSPY protein in both intracellular and cell surface fractions corresponding to the respective intracellular and cell surface markers. Bottom right showed similar western blot analysis with Group-2 mouse sera. Red arrows indicate the respective TSPY band, despite in small quantity, on the cell surface fraction

We postulate that TSPY protein/fragments/peptides were present on the surface of the positive tumor cells, probably through two likely mechanisms: TSPY protein/fragment mislocalization [55, 56] or TSPY peptide/MHC-I (TSPY pMHC-I) complex formation [57–59] on the cell surface, which could activate B cells for plasma cell differentiation and specific antibody production and cytotoxic T cells in eliciting the humoral and cellular immune responses respectively in the killing of positive tumor cells [60, 61]. To determine if TSPY protein/fragments could be located on the cell surface [55–58, 62], we had conducted a flow cytometry analysis of the human HCC tumor HuH7 cells, which do not harbor a Y chromosome nor TSPY gene [63], transfected with either a TSPY gene or control vector alone, representing TSPY-positive and negative tumor cells respectively. Flow cytometry for *surface* protein on live cells [64] was performed on respective HuH7 cell populations (Fig. 5A) with a panel of 9 monoclonal antibodies previously generated against purified TSPY recombinant protein [65]. The results showed that these monoclonal antibodies bound to epitopes on the cell surface of TSPY-positive HuH7 cells with variable degrees of affinity (Fig. 5A, red = TSPY positive and blue = TSPY negative cells). Importantly, among the effective monoclonal antibodies, the reactive domains of clones #7 and #22 were previously mapped to the C- and N-terminus respectively (Fig. 5B), suggesting potential multiple epitopes on the TSPY protein on the cell surface of HuH7 cells. Since the monoclonal antibodies were initially generated with purified TSPY protein [65], we surmise that they could recognize TSPY protein/fragments mislocalized on the surface of these tumor cells [55, 56]. To confirm such postulation, cell surface and intracellular proteins of the TSPY-positive HuH7 cells were fractionated with the Pierce Cell Surface Biotinylation and Isolation kit [66] and analyzed with western blots using the TSPY rabbit polyclonal antibodies and positive mouse sera. The results confirmed the presence of TSPY protein, abide in smaller quantity compared to intracellular fraction, on the cell surface of positive HuH7 cells (Fig. 5C), supporting the notion that TSPY protein/fragment could be mislocalized on the cell surface of positive cells.

### Cellular immune responses to TSPY-positive tumor cells involves MHC-I complexes on the cell surface

**Fig. 6:**
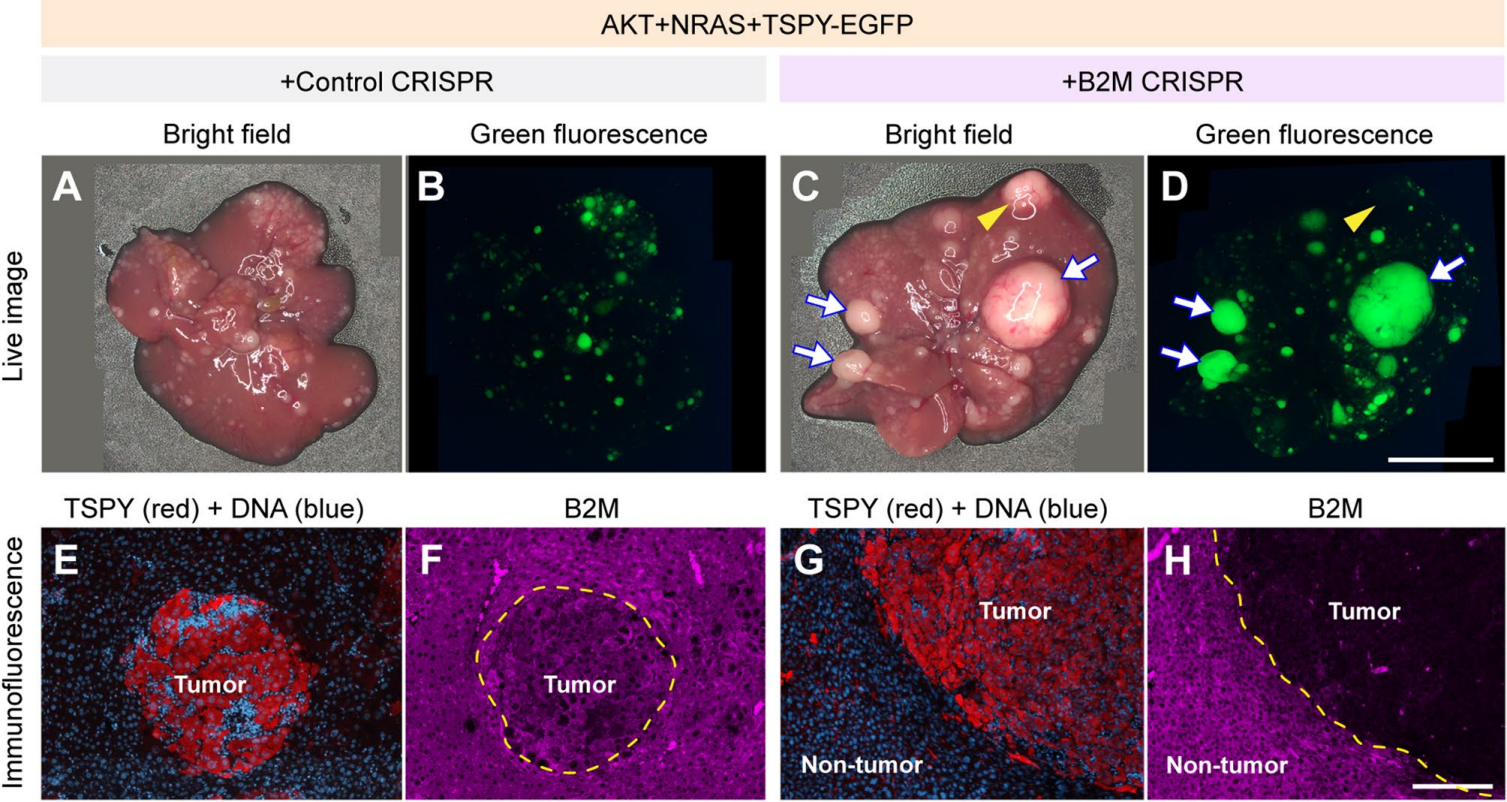
Involvement of MHC-I complex in the cellular immune elimination of tumor cells. The MHC-I complexes are important for presentation of self and non-self entities of cellular proteins on the surface and responsible for cellular immune responses to antigenic cancer-testis antigen, i.e. TSPY. Using a CRISPR strategy to knockout a key component, i.e. β2-microglobulin (B2M), of the MHC-I complex, such peptide-MHC-I complex formation and cellular immune responses could be minimized/eliminated. Tumors derived from AKT + NRAS + TSPY-EGFP tumors co-injected with a non-specific (control) sgRNA in the CRISPR procedure showed only small tumor foci (**A** & **B**), which were positive for both TSPY and B2M protein (**E** & **F**), suggesting they could be subjected to cellular immune responses and elimination. Those injected with specific sgRNA (**C** & **D**) showed two types of large tumor foci in addition to the small foci. One minor type of large tumor foci was negative for both TSPY and the co-expressed EGFP (**C** & **D**, yellow arrowheads), representing no TSPY expression in the tumor cells. The others represented the major type of large tumor foci (white arrows), showing positive for TSPY and the co-expressed EGFP (**D** & **G**) but negative for B2M (**H**). Thus, these large tumor foci escaped immune elimination by cytotoxic T cells through either deleting/suppressing the antigenic TSPY protein expression or minimizing TSPY peptide-HMC-I complex formation and cell surface presentation in the absence of b2-microglobulin (B2M). Scale bar = 1 cm in A-D; 200 μm in E-H

The MHC-I molecules are expressed in all nucleated mammalian cells including hepatocytes; they form complexes with short peptides of 8–12 amino acids from the cellular proteins and present them on the cell surface as a mechanism for identifying self versus non-self entities of cells in the immune microenvironment [67, 68]. As evidenced from the western blots (Fig. 4), TSPY is degraded/processed to fragments/peptides, which could form MHC-I complexes, be presented on the cell surface and be recognized as non-self antigens, thereby activating cellular immune responses from CD8^+^ cytotoxic T cells [59, 61, 69, 70]. The β2-microglobulin (B2M) is an essential component for the MHC-I complex [59]. In the absence of B2M, such MHC-I complex formation and cell surface presentation will be abolished/compromised, thereby minimizing any cellular immunity actions to any foreign/tumor entities [71]. To address the possible involvement of TSPY peptide-MHC-I complex(es) and cell surface presentation in the immune elimination of TSPY-positive tumors, we had performed a genome editing experiment using the CRISPR-Cas9 strategy to knockout (KO) the mouse B2m gene in the hepatocytes of the host animals [32, 72] by co-injecting with either a plasmid expressing the Cas9 nuclease and a B2m-specific short-guide RNA (sgRNA) (Group-6, Table 1) or a control plasmid expressing the Cas9 nuclease and non-specific sgRNA (Group-7, Table 1). The results showed that the mice injected with control sgRNA harbored small green foci in their tumors (Fig. 6A & B) while those with the B2m specific sgRNA harbored heterogeneous mixture of large and small green foci (Fig. 6C & D). Immunohistochemistry showed that the small foci in the control tumors expressed both TSPY and B2M in the tumor cells (Fig. 6E & F). In the B2m KO mice, two types of large tumor foci were present: (1) large foci negative for EGFP fluorescence, representing no TSPY protein expression (Fig. 6C & D, yellow arrow heads) and (2) large green fluorescent tumor foci (Fig. 6C, D, white arrows) positive for TSPY, but negative for B2M protein (Fig. 6G & H). These results suggested that the B2m gene could be successfully knocked out in selected hepatocytes of the tumors in the host animals. The small tumor foci expressing both TSPY and B2m were capable of forming TSPY-peptide-MHC-I complexes on the cell surface and were targeted by the cytotoxic T cells and immune elimination. The large tumor foci could result from insufficient/absence of TSPY peptide-MHC-I complex formation, thereby escaping such cytotoxic T cell attacks and elimination. We surmise that the large non-fluorescent tumor foci originated from tumor cells without TSPY co-integration/expression with the oncogenes while those fluorescent ones expressed TSPY but lacked B2M protein, essential for peptide-MHC-I complex formation. Collectively, TSPY-MHC-I complex formation on the cell surface were abolished/minimized in these large tumor foci, thereby providing a tumor microenvironment for tumor evasion to the cytotoxic T cell immune attacks and elimination. These findings support the notion that TSPY peptide-MHC-I complexes on the cell surface could be partially responsible for cellular immune elimination of positive tumors in the host animals.

## Discussion

The TSPY gene was one of the early genes isolated from the human Y chromosome [73]. It had been mapped onto the critical region harboring the only oncogenic locus GBY on this male-specific chromosome [12]. Various studies had focused on its candidacy as the gene for GBY, results of which had indeed demonstrated that TSPY is capable of accelerating cell proliferation through its actions on the cyclin B-CKD1 kinase activities [19], essential for G_2_/M progression. The rapid transition of the G_2_/M could compromise the checkpoints at this stage of the cell cycle, potentiating genome instability and oncogenesis [23]. Thus, TSPY has been considered as a proto-oncogene on the human Y chromosome, capable of promoting oncogenic growth [20]. Further studies demonstrated that it is aberrantly expressed in various somatic cancers, including prostate cancer and hepatocellular carcinoma, among others [21, 22]. Such expression pattern thereby highlighted TSPY as a cancer-testis antigen likely involved in cell proliferation and genome instability contributing to the male biases in these somatic cancers. However, various transgenic strategies in establishing transgenic mouse models for studies of its roles in human cancers have only limited successes [74], except when the human transgene is integrated onto the mouse Y chromosome and is expressed in a similar pattern as that in humans, i.e. predominantly in the germ cell of the testis in the host animals [75]. Significantly, similar to the human situations, the Y-located human TSPY transgene could be activated under oncogenic conditions, such as in the background of a prostate cancer mouse model [76]. These results suggest that both Y chromosome location and testis-specific expression are essential criteria for the proper functions of this Y chromosome gene in both humans and mouse model. It is also worthwhile noting that the mouse Tspy gene on the Y chromosome is a pseudogene harboring various in-frame termination codons and is incapable of coding for any functional protein [77]. Hence, this Y-located TSPY transgenic mouse line is a significant mouse model of TSPY as a male-specific cancer-testis antigen [78].

**Fig. 7:**
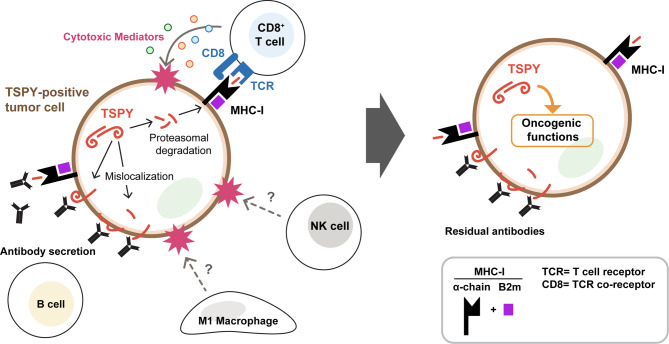
Dual functions of the male-specific TSPY cancer-testis antigen in hepatocellular carcinoma development. At early stage, the TSPY protein in positive tumor cells (left) could be mislocalized on the cell surface and stimulate B cell differentiation into antibody-secreting plasma cells and memory cells, thereby eliciting humoral immune responses in the elimination of TSPY-positive tumor cells. TSPY protein could also be degraded/processed into antigenic peptides by the proteasomes, form MHC-I complexes and be presented on the cell surface, resulting in activation of cytotoxic CD8^+^ T cell responses and elimination of positive tumor cells. Other immune cells, e.g. NK cells in innate immunity and M1 macrophages in inflammatory responses, could also be activated in the overall immune responses and elimination of TSPY-positive tumor cells. Residual tumor cells could acquire resistance to such immune responses or emerge from an immune exhaustive environment (right), in which the proliferative properties of TSPY could become effective, thereby promoting oncogenesis at late stage of hepatocellular carcinoma development

The current studies using the hydrodynamic tail vein injection strategy to generate TSPY-positive tumors in the liver has provided a critical window on revealing the immunogenic nature of the TSPY protein. Our results demonstrated that aberrant expression of TSPY protein elicits significant immune responses to and elimination of TSPY positive tumor cells in this mouse HCC model. Residual tumor cells could resume tumorigenic growth through either evasion of such immune responses or immune exhaustion in the tumor microenvironment at late stage [79]. We surmise that tumors with TSPY-mediated immune suppression at the early stage could resemble those of patients with low TSPY expression and good prognosis while those that resume tumorigenic growth could resemble those of patients with high TSPY expression and poor prognosis and survival [26, 30]. Although preliminary in nature, our TSPY transgenic mouse model offers a unique opportunity to further explore the role of this male-specific gene in various stages, such as cirrhosis, pre- and post-oncogenesis, and tumor progression to late stages of hepatic oncogenesis. Having a humanized TSPY transgenic mouse model [76] could offer opportunity for further exploration of its contributions to the liver disease and oncogenic processes. Our transcriptome studies clearly demonstrated that various immune and inflammatory responses and signaling pathways were activated in early stage of hepatic oncogenesis in TSPY positive tumors. Using specific biomarkers, we showed that different immune cell types involved in these immune and inflammatory processes were indeed activated at this stage of oncogenesis. Pairwise analyses of differentially expressed genes confirmed that such immune and inflammatory pathways were specific for TSPY-positive tumors at early stage. Importantly, we demonstrated that TSPY protein could be mislocated on the cell surface and capable of activating the B cells to differentiate into antibody synthesizing and secreting plasma cells and activate the humoral immune responses [60] (Fig. 7, left). Further, using a transient CRISPR-mediated gene knocking out strategy on the mouse β2-microglobulin, we showed that TSPY could be processed/degraded through proteosomes, likely resulting in TSPY-specific peptides capable of forming MHC-I complexes on the cell surface of tumor cells, activating the CD8^+^ cytotoxic T cells and killing of the positive tumor cells [61]. We surmise that such cellular immunity contributed *partially* the overall immune responses to the TSPY-positive tumor cells. Thus, knocking out the β2-microglobulin in the tumor cells would eliminate TSPY-peptide MHC-I complex formation and presentation on the cell surface, thereby abolishing such CD8^+^ cytotoxic T cell attacks on these B2m negative but TSPY-positive tumor cells, which were then grew to larger tumor foci. These findings collectively suggested that both humoral and cellular immune responses, and likely other immune processes e.g. M1 macrophage and innate immune NK cells, were activated in the immune elimination of TSPY-positive tumor cells at the early stage of hepatocarcinogenesis. We showed that residual tumor cells could evade such immune surveillance and emerge as aggressive tumors at late stage (Fig. 7, right). Currently, we are uncertain if the evasion of immune surveillance by TSPY-positive tumor cells was caused by adaptation/evolution of the tumor cells or immune and inflammatory exhaustion in the tumor microenvironment [79, 80]. Nevertheless, the TSPY continuous expression could promote cell proliferation and aggressive tumor progression, leading to poor survival and outcomes at the late stage of HCC development (Fig. 7, right). The present HCC model offers means to examine the molecular mechanisms by which TSPY could mediate such immune elimination, escape the immune surveillance, and promote oncogenic growth at later stage of HCC. Further, as a cancer-testis antigen, TSPY protein possesses significant immunogenicity capable of eliciting robust immune and inflammatory responses and thus is a key molecule for development of immunotherapy for HCC, particularly personalized treatments for positive patients. HCC patients could be initially screened for the presence of TSPY autoantibodies in their circulation to identify those harboring and expressing TSPY protein [52]. Positive patients could be good candidates for treatments with TSPY-based immunotherapeutic strategies, including reactivation of the immune responses through immunization of a therapeutic cancer vaccine [81] or treatments with antibody-drug conjugates [82] and in combination of other therapeutics [83], thereby providing effective clinical management of this prevalent liver cancer among male patients.

## Electronic supplementary material

Below is the link to the electronic supplementary material.

Supplementary Material 1

Supplementary Material 2

Supplementary Material 3

## Data Availability

The data are presented as supplementary materials Table S2 and S3 of the article.

## Abbreviations

AR: androgen receptor.
B2M: β2-microglobulin.
CDK1: cyclin-dependent kinase 1.
CRISPR: clustered regularly interspaced short palindromic repeats.
CTA: cancer-testis antigen.
DAVID: Database for Annotation, Visualization and Integrated Discovery.
DSD: disorder of sex development.
EGFP: enhanced green fluorescent protein.
GBY: gonadoblastoma locus on the Y chromosome.
HCC: hepatocellular carcinoma.
MHC-I: major histocompatibility complex class I.
TSPY: testis-specific protein Y-linked.
TUNEL: terminal deoxynucleotidyl transferase dUTP nick end labeling.
